# Functional properties of eyelid conditioned responses and involved brain centers

**DOI:** 10.3389/fnbeh.2022.1057251

**Published:** 2022-12-09

**Authors:** Gloria G. Parras, Rocío Leal-Campanario, Juan C. López-Ramos, Agnès Gruart, José M. Delgado-García

**Affiliations:** Division of Neurosciences, University Pablo de Olavide, Seville, Spain

**Keywords:** claustrum, cerebellar interpositus nucleus, eyelid classical conditioning, facial motoneurons, hippocampus, motor cortex, medial prefrontal cortex, red nucleus

## Abstract

For almost a century the classical conditioning of nictitating membrane/eyelid responses has been used as an excellent and feasible experimental model to study how the brain organizes the acquisition, storage, and retrieval of new motor abilities in alert behaving mammals, including humans. Lesional, pharmacological, and electrophysiological approaches, and more recently, genetically manipulated animals have shown the involvement of numerous brain areas in this apparently simple example of associative learning. In this regard, the cerebellum (both cortex and nuclei) has received particular attention as a putative site for the acquisition and storage of eyelid conditioned responses, a proposal not fully accepted by all researchers. Indeed, the acquisition of this type of learning implies the activation of many neural processes dealing with the sensorimotor integration and the kinematics of the acquired ability, as well as with the attentional and cognitive aspects also involved in this process. Here, we address specifically the functional roles of three brain structures (red nucleus, cerebellar interpositus nucleus, and motor cortex) mainly involved in the acquisition and performance of eyelid conditioned responses and three other brain structures (hippocampus, medial prefrontal cortex, and claustrum) related to non-motor aspects of the acquisition process. The main conclusion is that the acquisition of this motor ability results from the contribution of many cortical and subcortical brain structures each one involved in specific (motor and cognitive) aspects of the learning process.

## Introduction

Since the late thirties of the past century (Hilgard and Marquis, 1935; Marquis and Porter, 1939), the classical conditioning of the nictitating membrane and/or the eyelid response has been used as a suitable experimental model for studying the function rules of this type of associative learning, the different brain sites involved in the acquisition, storage and retrieval of new motor abilities, and the neural and subcellular mechanisms underlying these processes (Gormezano et al., 1983; Yeo et al., 1985; Woody, 1986; Thompson, 1988; Welsh and Harvey, 1989a, 1991; Christian and Thompson, 2003; Delgado-García and Gruart, 2006; Manto et al., 2012; de Zeeuw and Ten Brinke, 2015; Takehara-Nishiuchi, 2018).

With regard to brain structures, cerebellar cortical and/or nuclear areas have been preferentially assumed to be the sites involved in the acquisition and/or performance of conditioned eyeblinks—a proposal not fully accepted by all researchers (Welsh et al., 1986; Welsh and Harvey, 1989a, 1991; Krupa et al., 1993; Gruart et al., 2000b; Christian and Thompson, 2003; Koekkoek et al., 2003; Perciavalle et al., 2013). Indeed, many other cerebral cortical and subcortical centers and circuits have been reported as involved in different aspects of the acquisition, storage, and extinction processes. For example, the intrinsic hippocampal circuit (Weiss et al., 1999; Múnera et al., 2001; Gruart et al., 2006), and the somatosensory (Leal-Campanario et al., 2006; Ward et al., 2012) and medial prefrontal (Weible et al., 2003; Siegel and Mauk, 2013; Caro-Martín et al., 2015) cortices have been proposed to be involved in this type of associative learning. In addition, subcortical structures such as thalamic nuclei (Sears et al., 1996; Bahro et al., 1999; Campolattaro et al., 2007), the amygdala (Boele et al., 2010; Sakamoto and Endo, 2010), the red nucleus (Sakamoto and Endo, 2010; Pacheco-Calderón et al., 2012), the striatum (Blázquez et al., 2002) and the claustrum (Reus-García et al., 2021) have been shown to participate in the generation of conditioned eyeblinks.

More recent proposals have suggested the joint, but specialized, involvement of cerebellar (cortex, nuclei), cortical (hippocampal, motor, prefrontal), and subcortical (amygdala, striatum, claustrum, red nucleus) structures in the different aspects (motor, attentional, cognitive, stimulus salience, associative strength, etc.) of classical eyeblink conditioning (Siegel et al., 2012; Caro-Martín et al., 2015; Ammann et al., 2016; Reus-García et al., 2021). The present review is aimed at addressing these questions considering the neural substrates of eyeblink conditioned responses (CRs) as a distributed system involved in the diverse functions present in eyeblink conditioning as a whole. Unless otherwise indicated, and for the sake of homogeneity, most of the experimental studies commented on here were carried out with unitary recordings in alert behaving rabbits and cats during the acquisition of a classical conditioning task using the same delay paradigm: a 350-ms tone as conditioned stimulus (CS) coterminating with a corneal air puff (100 ms) as the unconditioned stimulus (US). Given the diversity of brain structures that have been implicated in classical eyeblink conditioning, we have focused our review on three structures related to the motor aspects of the generated CR (motor cortex, red nucleus, and cerebellar interpositus nucleus) and on three other brain structures preferentially related to attentional and cognitive aspects of the learning process [hippocampal intrinsic circuit, medial prefrontal cortex (mPFC), and the claustrum]. Interestingly, these structures have been identified in different functional imaging studies in humans (Molchan et al., 1994; Blaxton et al., 1996).

## Functional Peculiarities of The Eyelid Motor System

Facial and extraocular muscles are originated from the branchial arch (Noden and Francis-West, 2006) and exhibit many physiological peculiarities, making them quite different, functionally, from skeletal muscles. To start with, the eyelid motor system has an almost negligible mass, is load-free, and according to the available data, is free of proprioceptors; as a result, it is devoid of a true stretch reflex (Porter et al., 1989; Trigo et al., 1999a). That means that eyelid responses are executed without any sensory feedback arising from the motor action. Thus, nictitating membrane/eyelid responses are an open-loop, a term indicating that the sensorimotor loop is not closed by any sensory feedback (Wolpert and Flanagan, 2016). These functional peculiarities of blink motor responses have important consequences in the neural organization of eyelid motor commands.

Nevertheless, and in spite of the blink’s apparent simplicity, there are different and precise reflexes, voluntary and classically conditioned movements that can be conducted with the eyelids. From a functional point of view, a blink can be defined as a reflex eyelid response evoked by the mechanical activation of corneal and periorbital skin mechanoreceptors (Kugelberg, 1952; Evinger et al., 1991; Gruart et al., 1995). Blinks also can be evoked by fast and intense acoustic and visual stimuli (Evinger and Manning, 1993; Gruart et al., 1995). The kinematics of reflex blinks is the result of the fast contraction of both orbicularis oculi muscles, also involving the cocontraction of the retractor bulbi muscle in those species possessing a nictitating membrane (Baker et al., 1980). Apart from participating in reflexively evoked blinks, eyelids are also involved in spontaneous blinks (aimed in part at corneal wetting) and in emotionally related responses such as winking and smiling in humans, and grimacing and friendly displays in felines (Bateson and Turner, 1988).

**Figure 1:**
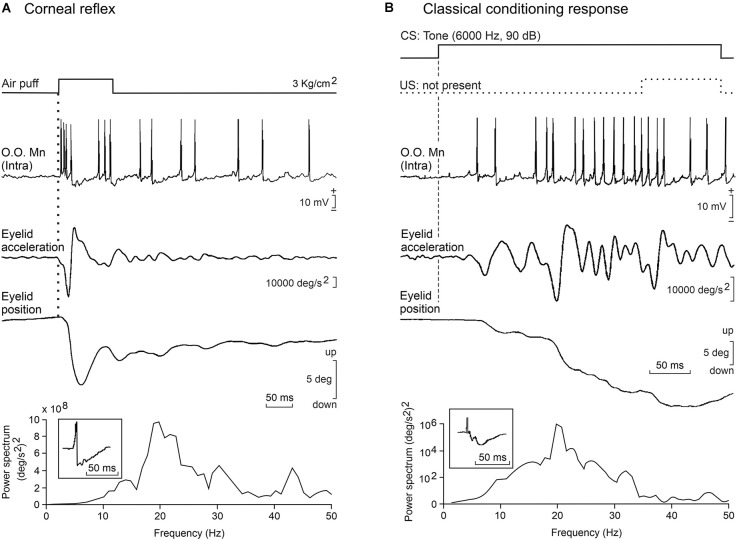
Basic firing properties of facial motoneurons. **(A)** A representative example of the phasic firing activity of an identified orbicularis oculi facial motoneuron recorded in a behaving cat during the presentation of an air puff to the ipsilateral cornea. From top to bottom are illustrated the stimulus presentation, the partial intracellular recording, and eyelid acceleration and position. Below is presented the power spectrum of lid acceleration trace with a dominant peak at 20 Hz, and in the inset is illustrated an average of the neuronal recording to show the duration of post-spike hyperpolarization (≈50 ms). **(B)** Tonic firing activity of the same motoneuron during classical conditioning in a well-trained cat. In the illustrated example, for a better observation of the typical firing during the eyelid conditioned response (CR), the air puff was not presented. Note that eyelid acceleration was only half that during the reflex response and that in this case motoneuron activity was mainly related to eyelid position, but the dominant oscillation of the CR and motoneuron hyperpolarization were similar to the profiles presented during the corneal reflex. Illustrated recordings are reproduced with permission and adapted from Trigo et al. (1999b).

A relevant example of timed and precisely elaborated eyelid responses is the generation of classically conditioned eyelid responses. For the past 90 years, the nictitating membrane/eyelid response has been used as an excellent experimental model to study the neural substrates underlying associative learning (Marquis and Porter, 1939; Gormezano et al., 1983; Woody, 1986; Welsh and Harvey, 1991; Christian and Thompson, 2003; Delgado-García and Gruart, 2006; Manto et al., 2012; de Zeeuw and Ten Brinke, 2015; Takehara-Nishiuchi, 2018). The kinematics and profiles of eyelid CRs are quite different from those presented by reflex blinks (Figure 1A), suggesting a different neural generation (Welsh, 1992; Gruart et al., 1995; Domingo et al., 1997; Trigo et al., 1999b). For example, while (in the cat) reflex blinks reach peak angular velocities of up to 2,000 deg/s to evoke a fast closing of the eyes, CRs never reach more than 200 deg/s and present a ramp-like closing of the eyelids (Figure 1B).

## Firing Characteristics of Orbicularis Oculi, Retractor Bulbi, and Abducens Motoneurons

A proper determination of the functional properties of brainstem motoneurons involved in voluntary, reflex, and classically conditioned eyelid responses is a necessary requisite for a better understanding of collected motor responses. We have studied the properties of these three types of motoneuron in alert behaving cats (Trigo et al., 1999b). The three types of recorded blink-related motoneurons (orbicularis oculi, abducens, and accessory abducens) were identified by their antidromic activation from their innervating muscles and/or their projecting axons. During the corneal reflex, both orbicularis oculi and accessory abducens motoneurons fire a phasic, double burst of action potentials (4–6 and 10–16 ms in latency) in response to air puffs presented to the cornea (Figure 1A). In cats, only orbicularis oculi motoneurons seem to fire in response to tone and/or flash presentations (Trigo et al., 1999b).

The activity of the orbicularis oculi muscle during the generation of eyelid CRs took place in a more tonic and gradual manner (Figure 1B). To start with, some putative excitatory postsynaptic potentials were observed by making intracellular recordings in orbicularis oculi motoneurons during the CS-US interval (Trigo et al., 1999b). Then, by the 2nd-3rd conditioning sessions, single action potentials were observed in facial motoneuron traces. The number of action potentials increased with training, reaching a tonic (≈30–40 spikes/s) firing by the 4th-5th sessions. By that time, clearly defined eyelid conditioned responses could be observed in the electromyographic activity of the orbicularis oculi muscle and/or in eyelid position profiles recorded with the magnetic search coil technique. It is important to notice that while rabbit accessory abducens motoneurons and the innervated retractor bulbi muscles are active during the performance of CRs (McCormick et al., 1982), no similar activity was observed in conditioned cats, probably because the latter animals need a much stronger stimulation to depolarize these rather big motoneurons (Trigo et al., 1999b).

Facial motoneurons present some bistable functional properties because their firing was linearly related to lid velocity during reflexively evoked blinks, but to eyelid positions during the performance of conditioned responses. These results are clearly indicative of the different neuronal origins and encoding of these two types of eyelid motor commands (Figure 1). Thus, the typical position profiles presented by reflex vs. conditioned eyelid responses are the result of the biomechanical properties of the orbicularis oculi and retractor bulbi muscles as well as of the membrane properties of their innervating motoneurons (Trigo et al., 1999b).

**Figure 2:**
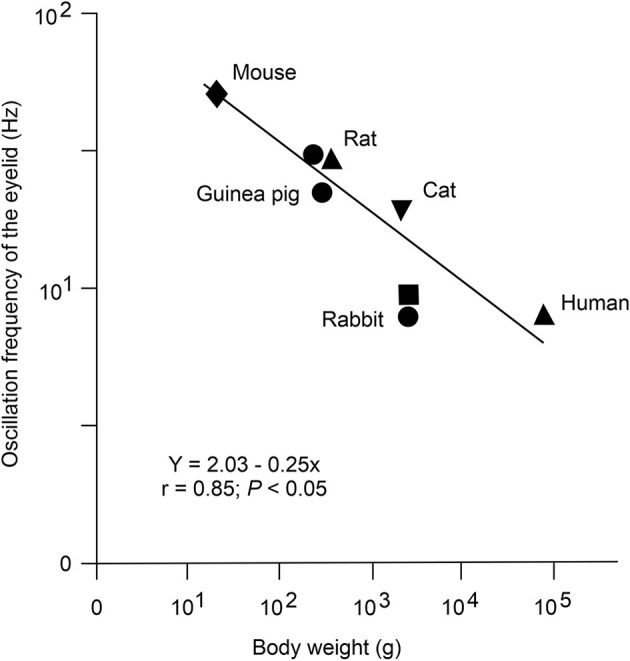
Presence of an inverse logarithmic relationship between the mean body weight and the dominant oscillation frequency of reflex and conditioned eyelid responses in distinct species of mammals. Data were collected from the following sources: (i) dots, from Gruart et al. (2000a); (ii) upward triangles, C. Evinger personal communication; (iii) downward triangle, from Domingo et al. (1997); (iv) square, from illustrated data in Welsh (1992); and (v) diamond, from illustrated data in Koekkoek et al. (2003). The same inverse logarithmic relationship between heart rate and body mass for mammals was reported years ago by Stahl (1967). Reproduced with permission and adapted from Gruart et al. (2000a).

The power spectra of both reflex and conditioned eyelid responses presented a dominant peak of ≈10 Hz in rabbits (Gruart et al., 2000a; Ammann et al., 2016) and of ≈20 Hz in cats (Gruart et al., 1995; Trigo et al., 1999b). Previous studies also noted the presence of oscillations in eyelid position traces or in the electromyographic activity of the orbicularis oculi and/or retractor bulbi muscles (Welsh, 1992). Those oscillatory activities are tuned to the size and viscoelastic properties of the corresponding eyelids, as determined in different species of mammals (Gruart et al., 1995, 2000a; Domingo et al., 1997; Koekkoek et al., 2002; Figure 2).

The wavy appearance of reflex and conditioned eyelid responses (easily noticed in eyelid position traces recorded with the magnetic search coil technique; see Figure 13 in Trigo et al., 1999b; and Figure 7 in Ammann et al., 2016) is also the result of the firing properties of facial motoneurons. For example, the membrane potential of orbicularis oculi motoneurons oscillated at ≈20 Hz following supraorbital nerve stimulation in behaving cats. This oscillation was probably the result of the intrinsic (the spike motoneuron afterhyperpolarization lasting ≈50 ms, and its later depolarizations) and the extrinsic properties of the circuits involved in eyelid blinks (Trigo et al., 1999b).

## The Red Nucleus Is Not A Mere Relay Center for Eyelid Conditioned Responses

**Figure 3:**
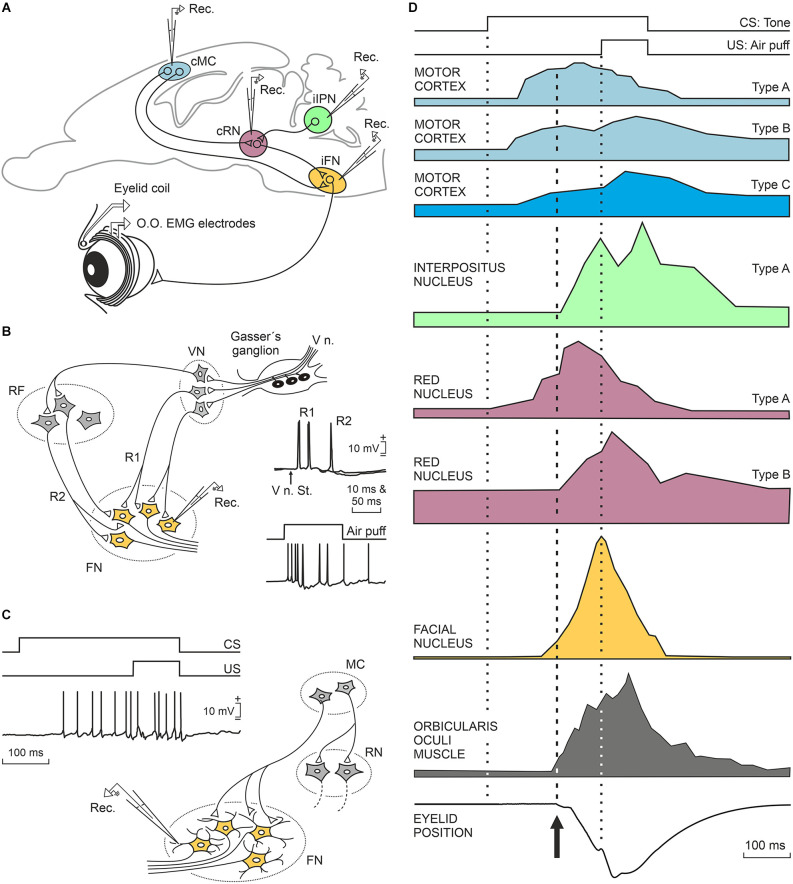
Characteristic firing profiles of neurons located in the motor cortex, cerebellar interpositus nucleus, red nucleus, and orbicularis oculi motoneurons during the performance of eyelid CRs in well-trained rabbits. **(A)** A diagram illustrating the location of these four motor-related areas and nuclei. Abbreviations: cMC, contralateral motor cortex; cRN, contralateral red nucleus; iFN, ipsilateral facial nucleus; iIPN, ipsilateral interpositus nucleus; O.O., orbicularis oculi muscle; EMG, electromyographic activity of the O.O. muscle; Rec., unitary recordings. **(B)** A representative diagram illustrating the basic neural circuit responsible for the corneal reflex, and partial intracellular recordings of identified O.O. motoneurons activated by the electrical stimulation of the supraorbital nerve (V n. St.) or by a corneal air puff. Abbreviations: V n., fifth nerve; VN, trigeminal nucleus, FN, facial nucleus RF, reticular formation; R1, R2, the two typical components of the corneal reflex (Kugelberg, 1952). The illustrated partial intracellular recording corresponds to an O.O. motoneuron recorded during a corneal air puff in a well-trained cat. **(C)** Proposed circuit underlying the generation of eyelid conditioned responses. The illustrated partial intracellular recording corresponds to an O.O. motoneuron recorded during CS-US presentations in a well-trained cat. Neuronal recordings illustrated in **(B,C)** were collected and modified by Trigo et al. (1999b). **(D)** At the top, is illustrated the delay conditioning paradigm used in these experiments. Below are shown profiles redesigned from neurons recorded in the motor cortex (rabbits, Ammann et al., 2016), interpositus nucleus (rabbits, Parras et al., 2021), red nucleus (rabbits, Pacheco-Calderón et al., 2012), facial nucleus (cats, Trigo et al., 1999b), and from the electromyographic activity of the O.O. muscle and the corresponding eyelid position (rabbits, Ammann et al., 2016). Dotted lines indicate CS and US presentations, while the dashed line indicates the beginning of the CR. CS, conditioned stimulus; US, unconditioned stimulus; CRs, conditioned responses.

The diagram illustrated in Figure 3A shows the location and main connections of the three main brain sites (red nucleus, cerebellar interpositus nucleus, and motor cortex) involved in the generation of conditioned eyelid responses and their relationships with the facial motor nucleus. The functional properties of these three brain sites will be described in the following three sections based on electrophysiological studies carried out in alert behaving rabbits.

The red nucleus is a mesencephalic premotor center mainly related to the organization of motor behaviors including face movements. The appearance of a red nucleus and of the corresponding rubrospinal tract is related to the presence of limb-like structures, as in the case of most terrestrial vertebrates and of certain species of rays (ten Donkelaar, 1988; Basile et al., 2021). The red nucleus receives dense projections from the sensorimotor cortices and cerebellar nuclei (Miller and Gibson, 2009; Basile et al., 2021). From a classical point of view, and mostly based on transient or permanent lesion studies (Chapman et al., 1990; Clark and Lavond, 1993), the red nucleus was assumed to participate in the neural circuit connecting the cerebellar interpositus nucleus with the facial and accessory abducens nuclei as a mere relay structure, since the acquisition and storage of acquired eyelid CRs would take place in the cerebellar cortex and/or nuclei (Krupa et al., 1993; Bracha et al., 2009; Freeman and Steinmetz, 2011). Given these initial concepts, it might be expected that not so many behavioral studies would be aimed at recording the activity of red nucleus neurons during the acquisition of classically conditioned eyelid responses (Desmond and Moore, 1991; Porras-García et al., 2010; Pacheco-Calderón et al., 2012). Although it was initially assumed that rubral projections to the facial and accessory abducens nuclei were mere collaterals of descending rubrospinal projections, more-specific neuroanatomical tracing techniques demonstrated that rubral projections to facial and accessory abducens nuclei originated from specific dorsolateral subdivision of the contralateral parvocellular red nucleus (Ruigrok and Cella, 1995; Morcuende et al., 2002). More recently, it has been convincingly shown in rabbits that fluorogold injections into the facial nucleus label neurons located in the dorsolateral area of the parvocellular red nucleus (Pacheco-Calderón et al., 2012).

We will describe in some detail the firing peculiarities of red nucleus neurons projecting to the facial and accessory abducens nuclei of behaving rabbits (Pacheco-Calderón et al., 2012). Those authors described two types of red nucleus cells related to eyelid CRs in behaving rabbits, and both of them activated antidromically from the facial and/or accessory abducens nuclei. Type A cells located in the red nucleus were activated 10–40 ms in advance of CRs and presented a peak firing during the CS-US interval (type A, violet color; Figure 3D). Type B cells did not fire in advance of the beginning of CRs and displayed a peak of activity during US presentation (type B, violet color; Figure 3D). In fact, the firing profiles of type B cells were similar to those presented by motor cortex type C neurons, although with a longer latency with respect to CS presentation (see below). The mean firing rate of type A and B neurons increased across conditioning in parallel with the integrated electromyographic (EMG) activity of the orbicularis oculi muscle, but not with the learning curve, a fact suggesting their main involvement in some motor aspects of the acquisition process (Desmond and Moore, 1991; Porras-García et al., 2010). Lidocaine injections in the motor cortex reduced the number and amplitude of CRs, without affecting eyelid reflex responses, and also decreased the mean firing rates of red nucleus neurons related to eyelid responses (Pacheco-Calderón et al., 2012). When lidocaine was administered to the interpositus nucleus (Pacheco-Calderón et al., 2012), the effects were similar to those described for the injected motor cortex, but in this case, reflex eyelid responses (evoked by US presentations) were also significantly decreased, as previously reported (Welsh and Harvey, 1989b). The strong disfacilitation of red nucleus neurons evoked by interpositus inactivation explains the reported decrease in eyelid reflex and conditioned responses evoked by cerebellar acute lesions. In contrast, motor cortical lesions evoke a smaller and transient depression of red nucleus neurons, a fact that helps to maintain the presence of eyelid CRs in these lesions (Pacheco-Calderón et al., 2012).

As indicated above, the firing activities of red nucleus neurons are more related to the kinematics of eyelid CRs than to the acquisition process. In fact, there are many studies indicating that red nucleus inactivation has no significant effects on learning curve profiles (Robleto and Thompson, 2008; Bracha et al., 2009; Freeman and Steinmetz, 2011). Nevertheless, other authors (Miller and Gibson, 2009) proposed a more active role of red nucleus neurons during the acquisition of new motor activities. In this regard, and as reported by Pacheco-Calderón et al. (2012), the rapid recovery of red nucleus neurons after lidocaine inactivation of the motor cortex suggests the putative role of this structure in the case of motor cortex lesions.

## The Cerebellar Interpositus Nucleus Reinforces and Improves The Performance of Conditioned Eyelid Responses

The involvement of the cerebellar cortex and/or the underlying deep nuclei in the acquisition, storage, and retrieval of classically conditioned nictitating membrane/eyelid responses has been repeatedly proposed, sustained on different functional bases, experimental approaches, and theoretical proposals (Llinás and Welsh, 1993; Carey and Lisberger, 2002; Christian and Thompson, 2003; Manto et al., 2012; de Zeeuw and Ten Brinke, 2015; Cheron et al., 2016; Takehara-Nishiuchi, 2018). Specifically, not well-defined areas of the interpositus (anterior, posterior) nucleus have been postulated as particularly involved in this peculiar type of associative learning (Krupa et al., 1993; Mauk, 1997; Bracha et al., 2000). The use of retrograde transneuronal tracing with rabies virus in rats further confirmed the connections of specific interpositus nucleus areas with eyelid motor centers (Morcuende et al., 2002), a proposal corroborated with other retrograde transsynaptic tracers (pseudorabies virus) in rabbits (Gonzalez-Joekes and Schreurs, 2012), by pharmacological and lesion studies in cats and rabbits (Yeo et al., 1985; Krupa et al., 1993; Hardiman et al., 1996; Mauk, 1997; Bracha et al., 1999; Christian and Thompson, 2003; Freeman and Steinmetz, 2011), and with single-cell recordings in behaving cats (Gruart et al., 2000b).

Nevertheless, the specific contribution of the cerebellar cortex and/or nuclei to the classical conditioning of eyelid responses is still an open question. Thus, while different pharmacological and lesion studies suggest that the interpositus nucleus could be the site for the acquisition and storage of this experimental model of associative learning (Krupa et al., 1993; Mauk, 1997; Bracha et al., 2000), other groups have proposed that the cerebellum could be mainly involved in the proper timing and performance of eyelid CRs generated in different brain regions (Welsh et al., 1986; Welsh and Harvey, 1989a, 1991; Welsh, 1992; Gruart et al., 2000b; Delgado-García and Gruart, 2002; Seidler et al., 2002; Koekkoek et al., 2003; Parras et al., 2021). Interestingly, changes in the intrinsic membrane excitability of cerebellar neurons have also been described following classical eyeblink conditioning in mice (Titley et al., 2020) and rats (Wang et al., 2018).

In a long series of experiments, we have recorded the activity of identified cerebellar nuclei neurons in behaving cats (Gruart and Delgado-García, 1994; Gruart et al., 2000b; Jiménez-Díaz et al., 2004), rabbits (Parras et al., 2021), and mice (Porras-García et al., 2010; López-Ramos et al., 2018). In an initial study (Gruart and Delgado-García, 1994), and in coincidence with other descriptions (Chen and Evinger, 2006), we recorded the activity of cerebellar nucleus neurons during blinks evoked by corneal air puffs, light flashes, and tones. Recorded neurons were identified by their antidromic activation from their respective brainstem projection sites. Although neurons related to experimentally evoked blinks were found distributed across the three cerebellar nuclei, most recorded blink-related neurons were concentrated in the dorsomedial part of the posterior interpositus nucleus. Further studies demonstrated the presence in cats of two types of neurons related to reflexively evoked blinks and to classically conditioned eyelid responses (Gruart et al., 2000b; Jiménez-Díaz et al., 2004). Type A neurons projected to the contralateral red nucleus and presented a sustained firing during the CS-US interval in significant coincidence with the presentation of eyelid CRs (green color; Figure 3D). In fact, type A neurons increased their firing during all types of downward displacement of the upper eyelid, lagging eyelid movements (recorded with the magnetic search coil technique) by >10 ms. Interpositus type A neurons recorded in behaving rabbits (Parras et al., 2021) and mice (Porras-García et al., 2010; López-Ramos et al., 2018) presented similar firing properties to those described for cats, and also lagged at the beginning of eyelid CRs. The discharge rate of type A neurons was linearly related to eyelid angular position or velocity, with a wide range of values. In cats, but not rabbits, we noted the presence of a second type of neuron, named type B. Type B neurons presented a regular sustained firing rate that was inhibited during the CS-US interval. Interestingly, they were activated antidromically from the red nucleus and/or from the oculomotor complex. Firing activities of type B neurons have been interpreted as related to the maintenance of the tonic activity of levator palpebrae muscles and to their inhibition during classically conditioned and reflexively evoked blinks (Gruart et al., 2000b). However, their absence in rabbits represents a setback for this interpretation.

The experimental manipulation of the posterior interpositus area where eyelid-related neurons are located was helpful for a proper interpretation of their role in the acquisition of eyelid CRs (Jiménez-Díaz et al., 2004). The local administration of muscimol (a GABA A ionotropic antagonist) decreased the amplitude of both reflex and classically conditioned eyelid responses in behaving cats, while the microstimulation of the same area (at 20 Hz—i.e., the resonant frequency of cat eyelid responses) increased them. Importantly, neither the application of muscimol to the posterior interpositus nucleus nor its microstimulation modified the learning curve in trained cats, suggesting that the putative role of cerebellar circuits was related to the proper performance of neuromuscular elements controlling eyelid kinematics, but not to the acquisition process (Jiménez-Díaz et al., 2004). Using nonlinear association analysis and the time-dependent causality method with the unitary activity of identified orbicularis oculi motoneurons and posterior interpositus nucleus recorded in cats during classical eyelid conditioning, Sánchez-Campusano et al. (2009) concluded that the cerebellum plays a modulating-reinforcing role in this type of associative motor learning.

## The Motor Cortex Plays A Significant Role in The Proper Generation of Classically Conditioned Eyelid Responses

Although the motor cortex presents a distributed and repeated representation of facial muscles (Huang et al., 1988; Morecraft et al., 2001; Müri, 2016) and is generally accepted to be one of the main neural sites involved in the acquisition and proper performance of new motor abilities (Evarts et al., 1983; Doyon and Benali, 2005; Monfils et al., 2005; Brecht et al., 2013; Gloor et al., 2015; Hayashi-Takagi et al., 2015; Kaufman et al., 2015), it is amazing that the long list of cortical, subcortical, and cerebellar structures considered putative sites for the generation and/or storage of conditioned eyelid responses usually does not include the motor cortex and its directly related premotor circuits. In this regard, just a few seminal studies carried out in behaving cats (Aou et al., 1992; Birt et al., 2003), and two more-recent ones (Hasan et al., 2013; López-Ramos and Delgado-García, 2021) performed in genetically manipulated and wild-type mice, have addressed the participation of motor cortex neurons in this type of associative learning. Since this review is mainly devoted to the analysis of results collected from unitary recordings during the classical conditioning of eyelid responses, we will comment here in detail on the recent study carried out by Ammann et al. (2016) in behaving rabbits.

According to Ammann et al. (2016), motor cortex neurons were not activated during reflex blinks, but they presented a burst of action potentials preceding spontaneous eyeblink responses. The authors described three types of motor cortex neurons depending upon the moment at which they presented their peak activity during CS and US presentations. Type A neurons were activated ≈70 ms after CS presentation, presented their peak response during the CS-US interval, and their firing preceded the beginning of the CR by ≈95 ms (light blue; Figure 3D). Type B neurons were activated at ≈50 ms following CS presentation, presented two peaks of activity—one during the CS-US interval and the other during US presentation—and preceded the CR by ≈110 ms (medium blue; Figure 3D). Finally, type C neurons were activated ≈70 ms after CS presentation, presented a peak response during US presentations, and preceded CRs by ≈90 ms (dark blue; Figure 3D). Interestingly, while types A and B neurons were activated antidromically from the facial nucleus, type C cells were activated from the red nucleus. Their antidromic activation from distal brain sites guaranteed that these three types of projecting neurons were pyramidal cells and not local interneurons.

The latency of motor cortex neurons was linearly related to changes in the latency of CRs across training, and the integrated neural responses were also linearly related to integrated orbicularis oculi EMG responses across conditioning. It is important to note that the local inactivation of the motor cortex with lidocaine prevented the expression of CRs in well-trained rabbits. In addition, the electrical stimulation of the motor cortex at 10 Hz (the resonant frequency of eyelid responses in rabbits; Gruart et al., 2000a) evoked eyelid responses with profiles quite similar to CRs evoked by CS-US joint presentations (see Figure 7 in Ammann et al., 2016). As far as we know, the motor cortex is the only brain structure able to evoke CRs following its stimulation at the appropriate and timed frequencies.

Although it has been suggested that direct descending projections from the motor cortex to the facial nucleus are only present in catarrhine primates (Sherwood, 2005), Ammann et al. (2016) have reported the presence of significant numbers of labeled terminals of motor cortex pyramidal neurons in the dorsolateral subdivision of the nucleus—namely, where orbicularis oculi motoneurons are located. In addition, the antidromic activation of type A and B neurons from the same area of the facial nucleus and the convincing effects of motor cortex timed stimulation of eyelid responses are suggestive of a key role of the motor cortex in the generation of eyelid CRs. Indeed, Fanardjian and Manvelyan (1987) have reported the presence of postsynaptic effects of motor cortex stimulation on facial motoneurons in anesthetized cats.

As reported by Ammann et al. (2016), type A and B neurons fire well in advance of CR initiation. Since orbicularis oculi motoneurons start to fire ≈2–3 ms preceding the activation of the orbicularis oculi muscle (Trigo et al., 1999b), we have to assume a slow-building depolarization of facial motoneurons suggestive of cortical projections to distal dendrites (Figure 3C). This is in contrast to the short latencies of facial motoneuron activation during the induction of the corneal reflex (Baker et al., 1980; Shaw and Baker, 1985; Trigo et al., 1999b), indicative of direct projections of second-order trigeminal neurons on the somas of facial motoneurons (Figure 3B). The characteristic phasic firing of facial motoneurons during the corneal reflex vs. their tonic firing during the performance of eyelid CRs is also indicative of the different afferent organization of cortical vs. trigeminal projections onto facial motoneurons. These theoretical proposals also explain the different profiles and kinematics of reflex vs. conditioned eyelid responses (Trigo et al., 1999b; Gruart et al., 2000a).

## The Medial Prefrontal Cortex Plays Different Cognitive and Permissive Roles in The Acquisition and Performance of Classically Conditioned Eyelid Responses

**Figure 4:**
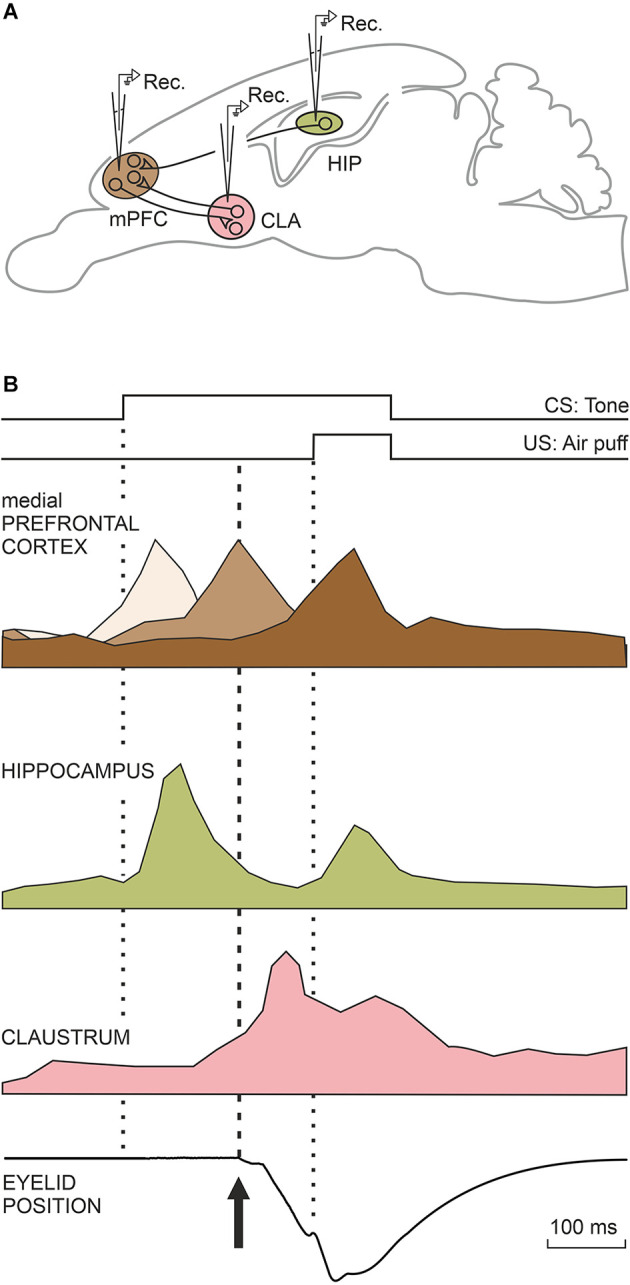
Characteristic firing profiles of neurons located in the mPFC, CA3, and CA1 areas of the dorsal hippocampus, and the claustrum during the performance of eyelid CRs. **(A)** A diagram illustrating the location of these four motor-related areas and nuclei. Abbreviations: CLA, claustrum; HIP, dorsal hippocampus; mPFC, medial prefrontal cortex. **(B)** The illustrated firing profiles were redesigned from representative mPFC (Caro-Martín et al., 2015) and claustral (type A; Reus-García et al., 2021) neurons recorded in classically conditioned rabbits and from hippocampal neurons (Múnera et al., 2001) recorded in conditioned cats. The three brown tones illustrating mPFC neurons indicate their different activation latencies. The eye-position trace was redesigned by Ammann et al. (2016). The dotted lines indicate CS and US presentations, while the dashed line indicates the beginning of the CR.

The functional activities of three main brain areas (mPFC, hippocampal intrinsic circuit, and claustrum) are illustrated in the diagrams depicted in Figure 4. These activities will be described in detail in the following three sections.

It is commonly accepted that the PFC occupies the highest hierarchical level in the functional organization of the many different cerebral cortical areas and that it deals with the adequate selection, timing, and execution of specific behaviors and the appropriate processing of cognitive information for decision-making tasks and contact interactions in social mammals (Fuster, 2008; Alexander and Brown, 2011; Carlén, 2017; Conde-Moro et al., 2019). In particular, the mPFC has been preferentially related to cognitive and emotional components of volitional and acquired behavioral abilities, including those dependent on classical (i.e., Pavlovian) and instrumental (i.e., operant) associative learning tasks (Powell et al., 1996; Corbit and Balleine, 2003; Weiss and Disterhoft, 2011; Jurado-Parras et al., 2012). While the activation of the rostral mPFC seems to inhibit the expression of both reflex and conditioned eyelid responses in behaving rabbits (Leal-Campanario et al., 2007), trace paradigms (also in rabbits) showed the caudal mPFC plays an important role in the acquisition and retrieval of eyelid CRs (Kronforst-Collins and Disterhoft, 1998; Powell et al., 2005). The caudal mPFC is also involved in the acquisition of classically conditioned eyelid responses during partial reinforcement and/or when a weak US is presented to the experimental animal (Powell et al., 1996; Kronforst-Collins and Disterhoft, 1998; Weible et al., 2003; Simon et al., 2005).

In an initial study (Leal-Campanario et al., 2013), we recorded the unitary activity of mPFC neurons during classical eyeblink conditioning using a delay paradigm. Recorded neurons were identified by their antidromic and/or synaptic activation from the mediodorsal thalamic nucleus; they were concentrated in the rostrodorsal part of the prelimbic cortex and on the anterior cingulate cortex. mPFC neurons presented activities related to the classical conditioning paradigm by the 4th conditioning session, reaching their peak activities by the 7th session. In the same experiment, we found that the electrical stimulation of the recording area decreased the percentage and amplitude of eyelid CRs, while the administration of a local anesthetic evoked the opposite effects.

In our previous study (Leal-Campanario et al., 2013) we observed that mPFC related to the conditioning process presented peaks of activity at different moments during the CS-US interval (350 ms, as was usual in this series of experiments), using a delay paradigm. This observation raised the question of the presence of a putative oscillatory activity in prefrontal neurons that could be related to the oscillatory properties of the eyelid motor system in rabbits or with cognitive processes aimed at determining the CS-US time intervals. These results prompted us in the following study (Caro-Martín et al., 2015) to record prefrontal neurons using CS-US intervals of different durations (50, 250, 500, 1,000, and 2,000 ms). In all of the cases, the CS coterminated with an air puff (100 ms) aimed at the animal’s cornea. The firing rate of each individual mPFC neuron presented a single, dominant peak during the CS-US interval, but the population as a whole presented peaks with a frequency dependent on the selected CS-US interval: ≈12 Hz for 250 ms, ≈6 Hz for 500 ms, and ≈3 Hz for 1,000 ms, suggesting the presence of a variable oscillator generating this type of oscillatory behavior (three shades of brown; Figure 4). No neural activity was collected for the shortest (50 ms) and longest (2,000 ms) CS-US intervals. The variability in the oscillation frequency presented by mPFC neurons was in contrast to that observed in eyelid reflex and conditioned responses (≈10 Hz), suggesting that the distributed timing of peak neural responses could be somewhat related to the determination of the CS-US time intervals. It is well known that very short (<50 ms) and long (>1,000 ms) CS-US intervals hinder or even prevent the acquisition of eyelid CRs. The well-defined oscillations presented by the rostral mPFC for 250-ms and 500-ms CS-US time intervals help to explain why these intervals seem to be optimal for the acquisition of eyelid CRs in rabbits (Gormezano et al., 1983; Gruart et al., 1995, 2000a).

In agreement with the above contentions, and apart from the permissive role of rabbit mPFC areas for the generation and expression of eyelid CRs (Leal-Campanario et al., 2007, 2013), the rostral mPFC neurons could play an important cognitive role in the determination of CS-US time intervals, with the help of the above-mentioned variable oscillator (Caro-Martín et al., 2015). These timing properties could also be related to the precise inhibition or activation of attentional or cognitive processes and selected sequential behaviors (Kolb et al., 2004; Fuster, 2008). Other authors have reported the presence of sustained firing (although presenting evident peaks and valleys in the illustrated firing profiles) in more-caudal and dorsolateral prefrontal areas (Weible et al., 2003; Weiss and Disterhoft, 2011; Siegel et al., 2012; Siegel and Mauk, 2013), indicative of the relevance and/or salience of the sensory information during the CS-US interval, and for the proper timing of the CR.

## The Hippocampus Determines The Salience of The Conditioned Stimulus Or The Strength of The Conditioned Stimulus-Unconditioned Stimulus Association

A basic conceptual tenet of contemporary neuroscience is that acquired motor and/or cognitive abilities are stored in the brain as functional and molecular changes in synaptic strength or efficiency (Ramón and Cajal, 1909–1911; Konorski, 1948; Hebb, 1949). Indeed, convincing relationships have been established between the acquisition of newly learned knowledge and the underlying changes in synaptic activities, as determined by genetic, molecular, and electrophysiological studies (Bliss and Collingridge, 1993; Kandel, 2001; Kishimoto et al., 2001; Neves et al., 2008; Wang and Morris, 2010). Nevertheless, it seems particularly important to determine changes in synaptic strength at the very moment of the acquisition process (Gruart et al., 2006; Whitlock et al., 2006). This *in vivo* approach was able to determine the sequential order in which synaptic changes are taking place, mainly regarding cortical circuits and whether activity-dependent synaptic changes in efficiency are related only to learning cues or also to the context in which learning tasks are being performed (Carretero-Guillén et al., 2015).

Of cortical structures, the hippocampus has received special attention, due to its definite neural and synaptic organization and because of its particular involvement in learning and memory processes as determined on the basis of clinical and lesion studies. The hippocampus has been related to the acquisition and retrieval of many different behavioral and cognitive functions, such as spatial orientation (Wang and Morris, 2010), object recognition (Clarke et al., 2010), and, importantly, classical conditioning of eyelid responses (Berger et al., 1983; McEchron and Disterhoft, 1997; Múnera et al., 2001).

Interestingly, two seminal studies reported changes in strength taking place in the perforant pathway-dentate gyrus (PP-DG) projection in rabbits (Weisz et al., 1984) and on the hippocampal CA3-CA1 synapse in mice (Gruart et al., 2006) during the acquisition of classical conditioning tasks. Apart from the two mentioned synapses, we included in our study (Carretero-Guillén et al., 2015) four additional hippocampal synapses (PP-CA3, PP-CA1, DG-CA3, and CA3-contralateral CA1). As indicated above, the aim was to determine whether synaptic changes in strength taking place across the hippocampal circuit are strictly limited to the acquisition process or are also related to the different contexts in which conditioning is taking place. For this, we activated the mentioned hippocampal synapses and recorded the evoked field excitatory postsynaptic potentials when rabbits were just sitting in the holding cage in the absence of any stimulus, during pseudoconditioning, and during trace and delay conditioning paradigms. Results indicated that context and pseudoconditioning training evokes in different (PP-DG, PP-CA3, and PP-CA1) afferent hippocampal synapses early and long-lasting changes that later disappeared across the successive sessions. Pseudoconditioning also evokes not-very-lasting changes in the efficacy of intrinsic (CA3-CA1 and CA3-contralateral CA1) hippocampal synapses. Interestingly, changes in synaptic strength during delay and trace conditioning sessions took place preferentially within the intrinsic hippocampal circuit (DG-CA3, CA3-CA1, CA3-contralateral CA1). These results clearly indicate that even for the acquisition of a rather elementary associative learning task, many different cortical (and surely subcortical) synapses could be involved, taking into consideration the task to be acquired and the context in which the learning is taking place (Carretero-Guillén et al., 2015).

Unitary recordings of CA3 and CA1 neurons were carried out in alert behaving cats (Múnera et al., 2001). Recorded pyramidal cells were identified by their antidromic activation from the ipsilateral fornix. Recorded hippocampal neurons followed the acquisition process, increasing their mean firing rates across conditioning sessions. Neurons fired when presented with either CS or US with a similar latency (≈75 ms), but with a larger response to the weak CS than to the strong US (pale green; Figure 4). Importantly, firing profiles and latencies (to CS and US presentations) of recorded neuron stimuli were similar, regardless of the different sensory modalities used as CS (tones, and airpuffs) or the different conditioning paradigms (trace, delay). In evident contrast, the evoked conditioned responses presented different latencies and profiles depending upon the CS used and the conditioning paradigm. Collected results clearly indicate that the firing of recorded pyramidal neurons does not encode eyelid parameters (such as position or velocity) for either reflex or conditioned eyelid responses. Using single-unit recordings in rabbits, Berger et al. (1983) and McEchron and Disterhoft (1997) described the presence of hippocampal neurons that displayed, as reported here, strong CS-evoked and weak US-evoked firing responses. A parsimonious interpretation of the reported results could be that the hippocampus is mainly involved in the determination of CS salience or predictive value (Rescorla, 1988) and/or the CS-US associative strength, but not in the generation of eyelid CRs. Classical studies carried out in humans and experimental animals (Thompson, 1988; Bechara et al., 1995; Clark and Squire, 1998) also indicate that the hippocampus is not necessary for the generation of CRs, in particular when evoked with delayed conditioning paradigms. Nevertheless, when trace conditioning paradigms are used, a hippocampectomy prevents the acquisition of eyelid CRs in rabbits (Moyer et al., 1990), and experimentally evoked long-term potentiation at the CA3-CA1 synapse also significantly decreases the percentage of CRs in behaving mice (Gruart et al., 2006). Apparently, the intrinsic hippocampal circuit needs to be functionally active during both the acquisition and the recall processes (Madroñal et al., 2016).

## The Claustrum Is Involved in Cognitive Processes Related to The Classical Conditioning of Eyelid Responses

Since the initial proposal of the claustrum as a landmark structure related to the functional integration of many different cortical and subcortical neural centers aimed at generating the conscious state (Crick, 1994), the number of proposals involving this peculiar brain structure in different cognitive-related functions has increased notably. Thus, and mostly based on neuroanatomical and hodological considerations, the claustrum has been proposed to participate in perceptual binding, the generation of internal cognitive states, and/or the integration of the different sensory modalities (Edelstein and Denaro, 2004; Crick and Koch, 2005; Mathur, 2014; Goll et al., 2015; Citri and Barretta, 2016; Jackson et al., 2018). More specifically, the claustrum has been proposed as the definite brain site where consciousness is generated (Crick and Koch, 2005; Kurada et al., 2019) or as the place for the segregation of attentive processes (Mathur, 2014; Goll et al., 2015; Atlan et al., 2018) and for salience detection (Smythies et al., 2012; Remedios et al., 2014; Smith et al., 2019).

The claustrum is a thin, laminar structure extending rostro-caudally between the insula and the putamen. In relation to its volume, the claustrum appears to be the most interconnected region of the brain (Torgerson et al., 2015). In accordance with its dense reciprocal connections, it could be assumed that the claustrum plays a significant role in neural activities related to associative learning. In this regard, it is well known that claustral neurons respond to stimuli corresponding to many different sensory modalities (Spector et al., 1974; Olson and Graybiel, 1980; Sherk and LeVay, 1981; Remedios et al., 2014). Specifically, we have recently studied in behaving rabbits the contribution of claustral neurons to the acquisition of eyelid CRs, paying particular attention to their relationships with cognitive vs. motor aspects of this well-known experimental model of associative learning (Reus-García et al., 2021).

Our unitary recordings were centered in the most rostro-dorsal part of the claustrum, a region related to somatosensory, motor, and prefrontal projection areas (Kowiański et al., 1997). Indeed, ≈70% of the recorded claustral neurons were activated synaptically from the ipsilateral motor cortex. In addition, ≈16% of them were also activated antidromically. The number of neurons activated from the mPFC was smaller, probably due to the rather large prefrontal area and the restricted areas of claustral neuron projections. We recorded two distinct types of claustral neurons, both of them related to the acquisition of eyelid CRs. Type A neurons started firing during the CS-US interval, but their firing extended up to 1 s after the US end (purple; Figure 4). Characteristically, type A neurons did not fire in response to single stimulus presentations. In fact, they only fired well after the initiation of eyelid CRs in well-trained rabbits. The discharge rate of type A neurons was not significantly related to the kinematic properties of CRs, such as the integrated area of the electromyographic activity of the orbicularis oculi muscle or the latency for CR presentations. In contrast to type A, type B neurons recorded during conditioning sessions decreased their firing rate during the CS-US interval. Their inhibition started slightly before the beginning of eyelid CRs. Type B cells did not present this typical inhibition in their firing rate during the presentations of single stimuli of different sensory modalities—namely, their firing inhibition was restricted to conditioning sessions in which eyelid CRs were already noticed. Both type A and B cells were particularly active during conditioning sessions presenting maximum rates of CRs, their presence decreasing afterwards. Interestingly, local field potentials recorded simultaneously in the claustrum and mPFC presented a significant comodulation in the delta and low gamma bands during the conditioning sessions in which the acquisition process took place (Reus-García et al., 2021).

In an additional experiment (Reus-García et al., 2021), we blocked the output of projecting claustral neurons with the vINSIST method (a virus-delivered inducible silencing of synaptic transmission). Blockage of claustral neuron output activities decreased the profile of the acquisition curve, without affecting the kinematic properties of evoked eyelid CRs. These results indicate that the claustrum was mostly involved in cognitive aspects of the acquisition process. Indeed, the electrical single and train stimulation of the rostro-dorsal claustrum did not evoke any type of eyelid response.

The above results clearly indicate that claustral neurons change their firing activities during the acquisition of eyelid CRs in rabbits. The characteristic properties of claustral neurons during conditioning indicate that they are more related to selected attentional and/or cognitive aspects of the acquisition process rather than to the kinematics and profiles of acquired CRs (Goll et al., 2015). Claustral neurons do not seem to be activated by single, and non-significantly relevant, stimuli of any sensory modality. In contrast, they are particularly activated by the paired CS-US presentations just at the moment of maximum change in the acquisition curve, right before reaching asymptotic values. The putative contribution of claustral neurons in the attentional and cognitive components of eyelid CRs has also been proposed in other learning tasks, such as those related to resilience to distraction (Atlan et al., 2018). It has also been reported that patients with attention deficit hyperactivity disorder show an increased activity in claustral circuits (Dickstein et al., 2006). It can thus be assumed that claustral cells present a precise inhibition-activation balance to deal with cognitive situations requiring recruiting attention.

## Conclusions

The present review has been mainly based on unitary recordings collected from behaving rabbits during the classical conditioning of eyelid responses using a delay conditioning paradigm. We have considered three well-defined brain areas (motor cortex, red nucleus, and cerebellar interpositus nucleus) clearly related to the generation and performance of eyelid CRs. Neurons located in the motor cortex and some neurons located in the red nucleus fire well in advance of the beginning of eyelid CRs, suggesting that they could have a causal relationship with the generation of these motor responses. In addition, the electrical stimulation of the motor cortex at the appropriate eyelid resonant frequency evokes eyelid motor profiles similar to those generated during CS-US presentations in well-trained rabbits. The red nucleus receives important afferents from the motor cortex and the interpositus nucleus and appears capable of substituting motor cortex functions when the cortex is experimentally disconnected. In contrast, the inactivation of the cerebellum evokes a profound depression of rubral neuron firing activities, explaining the typical depression of conditioned and reflex responses observed following acute cerebellar nuclear lesions. In opposition to what is described here for motor cortex neurons, cerebellar interpositus neurons fired in simultaneity with eyelid CR profiles, suggesting that they cannot be the site initiating these acquired CRs. Nevertheless, the experimental manipulation of interpositus neurons is able to modulate CR profiles and amplitudes without changing learning curve profiles, suggesting a major role of cerebellar circuits in the proper performance, timing, and modulation of acquired CRs. A definite demonstration of the relative contributions of these three brain areas to the generation and expression of eyelid CRs could be the simultaneous recording of the simultaneous unitary recording of the identified motor cortex, red nucleus, and cerebellar interpositus neurons in rabbits across a complete classical eyeblink conditioning task.

Three additional brain areas (hippocampal CA3 and CA1 areas, mPFC, and claustrum) are not directly related to the generation of the CR, but to different cognitive aspects also involved in the acquisition process, such as the salience or relevance of the CS across training, the CS-US associative strength (hippocampus), the proper determination of CS-US intervals (mPFC), and the attentional and cognitive processing of the acquired sensory association (claustrum). As recently shown (Reus-García et al., 2021) the mPFC and the claustrum are functionally interconnected and synchronize their local field potentials during the acquisition process. In addition, the dorsal hippocampus projects to the mPFC and are connected across the thalamus. Following some previous studies from our laboratory (Leal-Campanario et al., 2007; Caro-Martín et al., 2015), it can be proposed that the mPFC integrated cognitive-related features and plays a permissive role in the proper and timed release of eyelid CRs. Further studies are still necessary to support this proposed role of mPFC circuits.

In conclusion, the classical conditioning of eyelid responses requires the participation of many different brain centers, each one dealing with a different aspect of the motor and cognitive components of this type of associative learning task.

## Author Contributions

AG and JMD-G prepared the illustrations and wrote the first draft of the article. GGP, RL-C, and JCL-R revised the manuscript and contributed to its improvement. All authors contributed to the article and approved the submitted version.

## Conflict of Interest

The authors declare that the research was conducted in the absence of any commercial or financial relationships that could be construed as a potential conflict of interest.

## Publisher’s Note

All claims expressed in this article are solely those of the authors and do not necessarily represent those of their affiliated organizations, or those of the publisher, the editors and the reviewers. Any product that may be evaluated in this article, or claim that may be made by its manufacturer, is not guaranteed or endorsed by the publisher.
